# A Novel Metabolic Connectome Method to Predict Progression to Mild Cognitive Impairment

**DOI:** 10.1155/2020/2825037

**Published:** 2020-08-18

**Authors:** Min Wang, Zhuangzhi Yan, Shu-yun Xiao, Chuantao Zuo, Jiehui Jiang

**Affiliations:** ^1^Institute of Biomedical Engineering, School of Communication and Information Engineering, Shanghai University, Shanghai, China; ^2^Department of Brain and Mental Disease, Shanghai Hospital of Traditional Chinese Medicine, Shanghai, China; ^3^PET Center, Huashan Hospital, Fudan University, Shanghai, China; ^4^Shanghai Institute for Advanced Communication and Data Science, Shanghai University, Shanghai, China

## Abstract

**Objective:**

Glucose-based positron emission tomography (PET) imaging has been widely used to predict the progression of mild cognitive impairment (MCI) into Alzheimer's disease (AD) clinically. However, existing discriminant methods are unsubtle to reveal pathophysiological changes. Therefore, we present a novel metabolic connectome-based predictive modeling to predict progression from MCI to AD accurately.

**Methods:**

In this study, we acquired fluorodeoxyglucose PET images and clinical assessments from 420 MCI patients with 36 months follow-up. Individual metabolic network based on connectome analysis was constructed, and the metabolic connectivity in this network was extracted as predictive features. Three different classification strategies were implemented to interrogate the predictive performance. To verify the effectivity of selected features, specific brain regions associated with MCI conversion were identified based on these features and compared with prior knowledge.

**Results:**

As a result, 4005 connectome features were obtained, and 153 in which were selected as efficient features. Our proposed feature extraction method had achieved 85.2% accuracy for MCI conversion prediction (sensitivity: 88.1%; specificity: 81.2%; and AUC: 0.933). The discriminative brain regions associated with MCI conversion were mainly located in the precentral gyrus, precuneus, lingual, and inferior frontal gyrus.

**Conclusion:**

Overall, the results suggest that our proposed individual metabolic connectome method has great potential to predict whether MCI patients will progress to AD. The metabolic connectome may help to identify brain metabolic dysfunction and build a clinically applicable biomarker to predict the MCI progression.

## 1. Introduction

Alzheimer's disease (AD) is a neurodegenerative brain disease and the most common cause of dementia, affecting millions of individuals worldwide [1]. In the intermediate stage between healthy aging and AD, one had developed cognitive deficits that can be diagnosed as mild cognitive impairment (MCI) [2]. Yet MCI disease is very complex, manifesting in clinical and neuropathological heterogeneity. The development of MCI is so labile that some remain in stable stage for many years after diagnosis and even revert to normal cognition. Early diagnosis of whether MCI patients will progress into AD is a daunting challenge.

Currently, most diagnosis studies on MCI conversion are using neuroimaging to acquire the features among MCI groups and then consider these features as biomarkers to predict MCI progression. As a frequently used neuroimaging technique in clinics, ^18^F-fluorodeoxyglucose (FDG) positron emission tomography (PET) imaging has been employed to detect the progression from MCI to AD [3]. For instance, clinical studies have revealed that FDG-PET could capture the information of resting-state regional cerebral glucose metabolic rate and predicted progression from MCI to AD, and it is mainly due to FDG uptake is reduced in abnormal high cerebrospinal fluid amyloid-*β* concentrations [4–6]. Recently, as the studies for FDG-PET imaging has advanced, there are various predictive modeling that has been proposed. Previously, some studies had used voxel-wise or region-of-interest- (ROI-) wise quantitative metabolic measures to predict MCI patient's progress into AD [7–9]. However, the subtle difference between stable MCI and progression MCI causes it difficult to diagnose and require exquisite predictive modeling. Besides, some studies have explored the application of deep learning methods, such as convolutional neural network (CNN) and deep Boltzmann machine (DBM) [10–12]. Nonetheless, deep learning methods face the challenge that limited sample of FDG-PET images and inaccessible biomarkers could hardly reveal the neuropathological changes associated with MCI conversion. Specifically, a previous study evaluated different predictive models on the same FDG-PET images for reproducible evaluation among MCI patients, and the results showed that the accuracy of classification range between 67% and 83% [13]. Thus, the development of new predictive modeling is necessary to assist diagnosis in clinical assessments and provide higher performance to predict MCI progression.

Connectome-based predictive modeling (CPM) is a novel data-driven protocol for developing predictive models of brain-behavior relationships, which has addressed challenges in brain neurodegenerative disease [14, 15]. Brain connectivity characterizes by different brain regions and the relationship between paired regions and discloses the dynamic communication by neuronal activity. The brain network based on FDG-PET images is an exciting new opportunity to understand the neurological disorders and has been proved adept at analyzing the abnormalities in AD patients [16–18]. As a result, CPM has the potential to provide a novel predictive model for MCI conversion.

The aim of this study is therefore to combine CPM with glucose metabolic imaging to identify discriminative features for accurately classifying whether MCI patients will progress to AD. Specifically, we have two secondary aims: (1) verify whether CPM can be used as a novel feature extraction method in FDG-PET images and (2) evaluate the predictive performance of our proposed metabolic CPM in MCI groups.

## 2. Materials and Methods

### 2.1. Motivation

**Figure 1:**
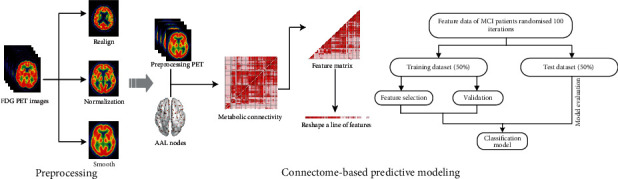
The overall framework of the proposed metabolic connectome-based predictive modeling (CPM) approach in this study.

The goal of this study is to develop a predictive model to capture predictive individual differences in MCI patients using CPM and FDG-PET imaging. We hypothesize that the combination of CPM and glucose metabolic imaging may identify the subtle brain metabolic dysfunction and use this information to obtain remarkable diagnosis performance. The framework of our proposed approach is summarized in Figure 1.

### 2.2. Participants and Imaging Protocols

The ^18^F-FDG-PET data used in this study were obtained from the Alzheimer's Disease Neuroimaging Initiative (ADNI) database (http://adni.loni.usc.edu). The primary goal of ADNI has been to test whether serial magnetic resonance imaging (MRI), positron emission tomography (PET), other biological markers, and clinical and neuropsychological assessments can be combined to measure the progression of mild cognitive impairment (MCI) and early Alzheimer's disease (AD). The institutional review board of ADNI approved all aspects of this study, and each participant has given written informed consent to undergo PET scanning of a long-term observational study.

This study acquired 420 MCI participants with FDG-PET scanning from ADNI-1, ADNI-2, and ADNI-GO database. The participant group was comprised of 242 stable MCI (sMCI) subjects and 178 progressive MCI (pMCI) subjects, and the images were used to establish a predictive model and test the validity of the model. The detailed eligibility criteria for all participants included the following: all participants underwent FDG-PET scanning and clinical cognitive evaluations at baseline visit and were followed during at least 36 months; stable MCI participants were diagnosed of MCI at baseline visit and did not progress to AD within 36 months of follow-up; and progressive MCI participants were diagnosed of MCI at baseline visit and progressed to AD within 36 months of follow-up. The demographic and clinical characteristics of all participants are summarized in Table 1.

Resting-state FDG-PET images at baseline visits were acquired and the detailed acquisition process could be found in the online information of ADNI. All participants were scanned using ^18^F-fluorodeoxyglucose (FDG) positron emission tomography (PET). There were 218 dynamic 3D images with six 5 min frames acquired 30 min after injection of 185 ± 18.5 MBq FDG. Besides, 202 participants were scanned with a static 30-minute acquisition.

### 2.3. Preprocessing

We preprocessed the brain FDG-PET images using the Statistical Parametric Mapping software (SPM12; Wellcome Department of Imaging Neuroscience, Institute of Neurology, London, UK) implemented in MATLAB (MathWorks Inc., Sherborn, MA). We realigned a time-series of FDG-PET images to generate a stable FDG-PET image. PET images were then spatially normalized into the Montreal Neurological Institute (MNI) brain space with linear and nonlinear 3D transformations. The normalized PET images were smoothed by a Gaussian filter of 8 mm full width at half maximum (FWHM) over a 3D space to blur individual variations in gyral anatomy and to increase signal to noise ratio for statistical analysis. Each PET image was intensity normalized to the global mean brain uptake to avoid individual uptake differences. For further analysis, the whole brain images were divided into 90 regions-of-interest (ROIs) defined by the automated anatomical labeling (AAL) atlas [19].

### 2.4. Metabolic Connectome Analysis

To acquire individual metabolic network from the FDG-PET image, we employed an individual-level graphical approach for metabolic connectivity, namely the Kullback-Leibler Divergence Similarity Estimation (KLSE) [20]. The globally normalized metabolic activity in ROIs was used to generate a glucose metabolic network for each participant. Firstly, the 90 cortical and subcortical ROIs derived by AAL were defined as network nodes. Then, for estimating the metabolic connectivity (metabolic correlations) between network nodes, we applied relative entropy into the spatial dimension, where the FDG-PET signal in ROIs reflected afferent synaptic activity and probability distribution between these ROIs denote interneuronal information transfer. The closer the relative entropy was to zero, the stronger metabolic connectivity between two random ROIs.

**Figure 2:**
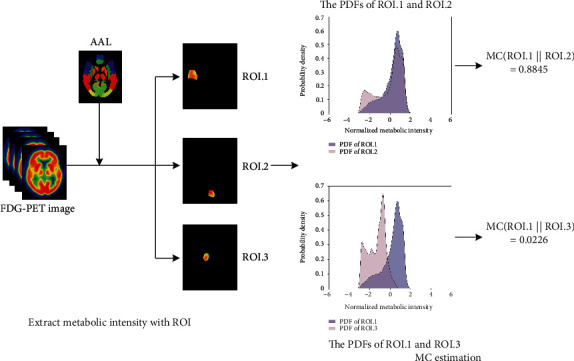
The framework for the estimation of metabolic connectivity (MC) between pairwise regions from individual FDG-PET image. Firstly, the FDG-PET image was divided into 90 ROIs, and the metabolic intensity values of voxels with random ROI were extracted. Then, kernel density estimation was employed to estimate the probability density function (PDF) of each ROI. Lastly, the KLSE algorithm was implemented to measure the metabolic correlation by the similarity among PDFs.

The detailed mathematical derivation of metabolic connectivity included the following three steps (see Figure 2). Firstly, we estimated the probability density function (PDF) of a random brain region (ROI) using a nonparametric way, namely the kernel density estimation (KDE). The estimation of kernel width was using a solve-the-equation bandwidth. Given the sample array quantifying the metabolic intensity of each voxel with ROI, we estimated the characteristic function as

(1) $$\begin{array}{c} \hat{\varphi }\left(\text{t}\right)=\left(\frac{1}{n}\overset{n}{\underset{j=1}{\sum }}e^{itx_{j}}\right). \end{array}$$

In this study, we had chosen the Gaussian function as a damping function to circumvent the question of diverging integral. After the damping function has been chosen, the Fourier transform formula may be applied, and the density estimation can be derived. Secondly, we assessed the metabolic correlation using the relative entropy between ROIs, which was estimated from the symmetric Kullback-Leibler (KL) divergence. The similarity of pairwise probability density functions (PDFs) was measured as given in the below mathematical equation:

(2) $$\begin{array}{c} D_{KL}\left(P\left\|Q\right.\right)=\int_{X}\left(P\left(x\right)\log\frac{P\left(x\right)}{Q\left(x\right)}+Q\left(x\right)\log\frac{Q\left(x\right)}{P\left(x\right)}\right)dx. \end{array}$$

in which *P* and *Q* represent the probability density functions PDFs of voxel intensities in pairwise ROIs. Lastly, t4he normalized similarity of these ROIs was acquired by KL divergence using the following representation:

(3) $$\begin{array}{c} KLS\left(P\left\|Q\right.\right)=\exp\left(-D_{KL}\left(P\left\|Q\right.\right)\right). \end{array}$$

Thus, a metabolic correlation matrix (90 × 90, region × region, 90 is the number of ROI) for each participant was obtained by the magnitude of KL-based similarity (KLS), where the correlation matrix elements represented the metabolic connectivity between pairwise nodes. Thus, we had constructed a metabolic network for each subject by AAL template (nodes) and KLSE algorithm (metabolic connectivity).

### 2.5. Predictive Modeling Analysis

For the metabolic network of each participant, a feature vector was obtained by extracting the lower triangular elements of correlation matrix. Each participant could acquire 4005 (90 × 89/2) features which defined as the metabolic connectivity between ROIs. The feature vectors then underwent predictive modeling analysis to discriminate sMCI and pMCI groups. To evaluate our proposed metabolic CPM approach fairly and minimize the influence factors, we employed three different classifiers, that are support vector machine (SVM), logistic regression (LR) model, and random forest (RF). Meanwhile, we also performed the Hosmer-Lemeshow goodness of fit test for the LR model.

To avoid the sampling variability of training and test datasets and obtain more stable estimates of predictive performance, we implemented the randomized cross-validation strategy. The detailed cross-validation procedures include two main steps. Firstly, all MCI participants were randomly partitioned into training dataset (50%, training the model) and test dataset (50%, test classification performance) for multiple times (100 iterations). Secondly, we implemented 10-fold cross-validation in the training dataset for hyperparameter optimization. In each random sampling of the training dataset, sparse regression least absolute shrinkage and selection operator (LASSO) approach was adopted to reduce the redundant features and to select the feature subset with higher discriminability [21]. The fitting models were built using those selected features. The predictive performance of models was evaluated by accuracy, sensitivity, specificity, the receiver-operating-characteristic curve, and the relevant area under the curve (AUC). The random split procedure was repeated for 100 iterations, and the mean and standard deviation of classification indicators were reported. SVM with linear kernel, L1-penalized LR model, random forest, and following receiver-operating-characteristic curve analysis and accuracy measurements were performed using the LIBSVM 3.23 toolbox and Statistics and Machine Learning Toolbox implemented in MATLAB.

### 2.6. Comparative Experiment

To further evaluate the performance of our proposed approach, two previous predictive methods in FDG-PET imaging were applied to the same predictive tasks: (1) the conventional feature quantification approach was performed based on mean metabolic uptakes in brain regions, and the FDG uptake values were regarded as features; (2) the spatial covariance analysis was performed on the training dataset to acquire a metabolic AD conversion-related pattern (ADCRP) topography, in which each voxel value represented the predictive weights [22]. The ADCRP expressions of each FDG-PET images were obtained by the *Z*-transformed score and regarded as the input features of the model. The comparative experiments underwent the same process procedures with the above metabolic connectivity.

### 2.7. Structural Brain Regions Associated with Effective Features

To verify the effectiveness of our proposed method, we further identified the structural brain regions associated with effective features. Firstly, part connectome features were considered efficient and discriminative features after undergoing the above feature select procedures. Then, the correlation matrix was generated which included only selected connectome features, and the weights of 90 brain regions were derived by column-summing the entries. In this case, the higher weight of regions implied that the region had more discriminative connectivity incident upon it. Lastly, we normalized the region weights by *Z*-score and sorted by the sign of the corresponding region weights. We obtained the structural brain regions associated with effective features by the criteria: *Z*-scored weight of region was greater than +1.0. These brain regions were considered relevant to AD progression [23].

## 3. Result

### 3.1. Clinical Characteristics

Clinical and demographic characteristics are reported in Table 1. The result of the age (*P* = 0.004) and the APOE *ε*4 positive rate (*P* < 0.001) showed significant changes between sMCI and pMCI groups. The cognitive assessments in the pMCI subjects tended to decrease than that in the sMCI subjects (Mini-Mental State Examination (MMSE), *P* < 0.001; Clinical Dementia Rating Sum of Boxes (CDRSB), *P* < 0.001; The Alzheimer's Disease Assessment Scale with 11 tasks (ADAS11), *P* < 0.001; and The Alzheimer's Disease Assessment Scale with 13 tasks (ADAS 13), *P* < 0.001). There were no significant differences in sex (*P* = 0.89) or education level (*P* = 0.496).

### 3.2. Predictive Modeling Analysis

**Figure 3:**
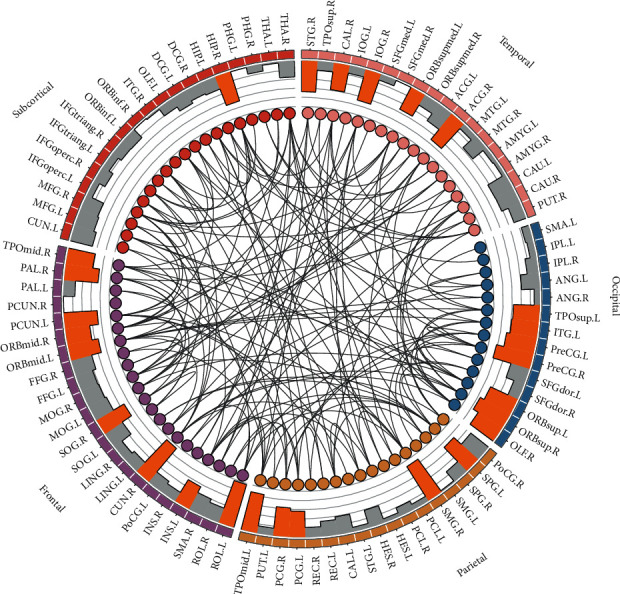
The brain metabolic connectivity associated with MCI conversion. The nodes in the circle represent 90 brain regions (ROIs). The different colors of nodes on the circle represent different anatomical classifications of brain regions (purple: frontal, orange: parietal, blue: occipital, pink: temporal, and red: subcortical). The black lines within nodes represent functional connectivity between ROIs selected with sufficient discriminative ability and associated with AD progression. The yellow histogram bars represent the hubs with more metabolic connectivity and the grey bars with little discriminative ability.

The 4005 functional connectivities drawn from the KLSE algorithm were the input features for predictive analysis. In the randomized cross-validations in 100 iterations, 153 metabolic connectivities were selected.  Figure 3 shows the brain metabolic connectivity associated with MCI conversion. The hubs were defined as the network nodes that had more quantitatively selected features. These hubs were highlighted to be associated with MCI conversion and mainly located in the brain regions including the precentral gyrus, precuneus, lingual, and inferior frontal gyrus.

We compared the predictive performances of different feature extraction methods: (1) ROI uptake; (2) MCI pattern; and (3) metabolic CPM.  Table 2 shows the results of different methods using three classifiers, and the best results for MCI groups diagnosis were achieved by the selected features from connectome with SVM classifier. The corresponding accuracy, sensitivity, specificity, and AUC values were 85.2%, 88.1%, 81.2%, and 0.933, respectively. Moreover, the CPM method achieved the lowest false-positive rate (18.8 ± 4.28%, 15.7 ± 6.64%, and 37.1 ± 7.48%) and false-negative rate (11.9 ± 3.17%, 19.1 ± 3.14%, and 12.4 ± 2.99%) in the three classifiers, respectively (Table 3). The receiver-operating-characteristic curves showed a high ability to diagnose MCI groups for metabolic CPM method (AUC, 0.933) but the lower discriminative ability for MCI pattern (AUC, 0.829) and ROI uptake (AUC, 0.831). The result of LR model showed that the information in the metabolic data has been extracted effectively and the goodness of the model fitting is high (*X*^2^ = 7.25, *P* = 0.51). Besides, the SVM classifier had better diagnostic ability than other classifiers (LR and RF) for the prediction of MCI conversion.

### 3.3. Discriminative Brain Regions Associated with MCI Conversion

**Figure 4:**
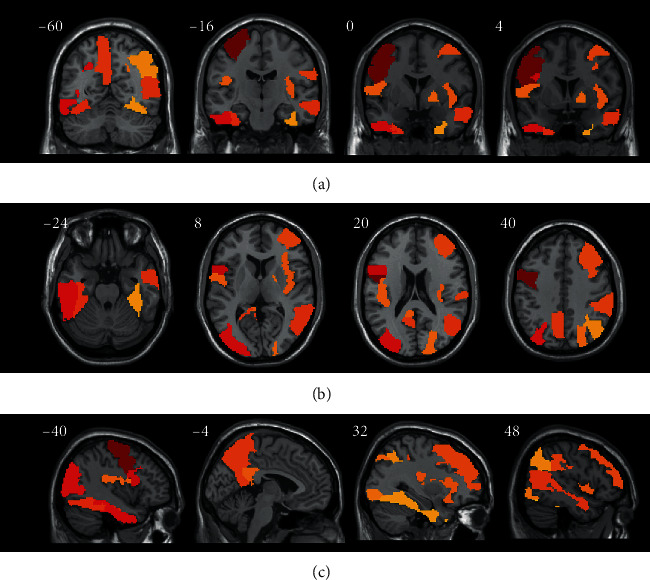
Topographic representations of connectome approach. The hubs regions were acquired from the training dataset using the LASSO approach in 100 iterations and were associated with the conversion from MCI to AD. The overlays are depicted in neurological coronal (a), transverse (b), and sagittal (c) orientations, respectively. Coordinates are displayed in MNI standard space.

We applied the concept of hubs into the brain regions associated with the progression from MCI to AD (Table 4). The result of hubs showed that some regions had more discriminative connectivity, including the precentral gyrus, precuneus, lingual gyrus, inferior temporal gyrus, and inferior frontal gyrus (Figures 4(a)–4(c)). When the PET images of pMCI participants were compared with sMCI participant's images, we observed significant hypometabolism in the precuneus, posterior cingulate, superior temporal gyrus, inferior frontal gyrus, etc. The result of metabolic CPM was implied that the hubs were statistically related to the conversion from MCI to AD.

## 4. Discussion

The accurate and sensitive diagnosis of MCI conversion is a paramount challenge to guide MCI patients for suitable clinical treatments as soon as possible. To address the challenge, in this study, we develop an efficiently metabolic CPM approach to diagnose whether MCI patients will progress to AD using metabolic images (^18^F-FDG-PET). The performance of our approach suggests that the metabolic connectivity derived by connectome analysis could be used for MCI conversion diagnosis and obtain excellent accuracy compared to other predictive models.

To further reveal the accuracy of our method, we compared the results between previous similar predictive methods and our proposed method, as shown in Table 5 [24–29]. Although the sample sizes and methodology of these studies are not identical, our proposed CPM method has better predictive performances with a large sample size. Maybe because of the deficiency of unbalanced sample sizes, the false-positive rates (FPR) and false-negative rates (FNR) of other methods are significantly higher than that of the results of our CPM model. Besides, previous studies summarize the MCI conversion approaches and propose a classification framework for reproducible and objective experiments using the ADNI database and other publicly available databases [13, 30]. The results show that the accuracy of these previous methods is ranging between 62% and 81% and the mean accuracy is 74%, and the reproducibility of these methods is not great. Meanwhile, our CPM method underwent a rigorous evaluation strategy to verify its feasibility and reproducibility. This method could capture more detailed and straightforward metabolic information within paired regions which performed as good as or better than many existing approaches. Thus, we believe that our metabolic CPM approach is more effective in predicting MCI conversion.

As an effective tool in the brain neuroscience field, CPM in AD has been pursued to develop an efficient biomarker for early diagnosis [14]. From the perspective of methodology, it is worth noting that the CPM approach is a generalizable model that takes brain connectivity data as input and generates predictions of MCI progression in novel subjects. In the neurodegenerative progress of AD, the brain changes involve the interaction of many brain regions rather than isolated regions, and neuronal degeneration is associated with cognitive deterioration. The between-region metabolic activities are impaired in MCI patients who are converting to AD. Thus, the most relevant indicator is the identification of corresponding brain regions and their connectivity. The brain network can delineate the full metabolic connectome and the connectivity dynamics of brain metabolism, and these findings provide opportunities to develop more accurate predictive models. The progressive disintegration of MCI patients is disclosed by the metabolic network. Therefore, the model based on selected connectivity has excellent diagnostic utility and reveals local brain pathologies.

From the results of connectome analysis, the metabolic connectivity abnormalities in MCI patients who converted to AD are mainly located in the precentral gyrus, precuneus, lingual, and inferior frontal gyrus. These brain regions are hubs of metabolic connectivity corresponding to synaptic disconnection. Metabolic connectivity in these regions decreases in progressive MCI patients compared to that in stable MCI patients. Previous MCI conversion investigation had shown the metabolic AD conversion-related pattern (ADCRP) which was characterized by relative decreases in temporoparietal, frontal, posterior cingulate, and precuneus cortex between sMCI and pMCI patients, and these results agreed with our experimental findings [22]. The voxel-wise two-sample *t*-test SPM analysis also found a highly similar metabolic difference which verified the pathophysiologic significance of regions that derived our approach [7, 31]. This result also indicates that our proposed metabolic CPM is an effective biomarker for MCI progression prediction.

## 5. Conclusion

In this study, we have proposed an innovative metabolic CPM method based on FDG-PET imaging, which can accurately diagnose whether the patients with MCI will eventually progress to AD. The experiment results suggest that metabolic connectivity can identify the metabolic abnormalities features and abnormal brain regions associated with MCI conversion. Our proposed metabolic CPM approach may be a potential tool with other clinical information to develop biomarkers for predicting the conversion of MCI patients.

## Figures and Tables

**Table 1:** Demographic information of all participants.

Group	sMCI (*n* = 242)	pMCI (*n* = 178)	*P* value
Sex (M/F)	136/106	102/76	0.89^a^
Age (year)	71.6 ± 7.82	73.7 ± 6.91	0.004^b^
Education (year)	15.9 ± 2.67	16.1 ± 2.65	0.496^c^
MMSE	28.2 ± 1.64	27.1 ± 1.80	<0.001^b^
CDRSB	1 (0.5,1.5)	1.5 (1, 2.5)	<0.001^c^
ADAS11	8.4 ± 3.4	13.0 ± 4.6	<0.001^b^
ADAS13	13.5 ± 5.5	21.0 ± 6.2	<0.001^b^
Conversion time (month)	\	20.6 ± 10.3	\
APOE *ε*4 positive rate	44.6%	68.5%	<0.001^a^

Data are given as mean ± standard deviation. *P*^a^: the chi-square test; *P*^b^: the two-sample *t*-test; *P*^c^: the Wilcoxon rank-sum test. MMSE: Mini-Mental State Examination; CDRSB: Clinical Dementia Rating Sum of Boxes; ADAS11: The Alzheimer's Disease Assessment Scale with 11 tasks; ADAS13: The Alzheimer's Disease Assessment Scale with 13 tasks; APOE *ε*4 positive rate: positive or negative for the presence of at least one *ε*4 allele.

**Table 2:** Predictive performance of different methods among MCI groups.

Classifier	Predictive method	Accuracy (%)	Sensitivity (%)	Specificity (%)	AUC
SVM	ROI uptake	74.8 ± 2.41	82.2 ± 1.72	66.7 ± 2.51	0.829 ± 0.035
	MCI pattern	76.7 ± 2.48	83.7 ± 4.46	67.1 ± 6.29	0.831 ± 0.026
	Connectome	85.2 ± 2.34	88.1 ± 3.17	81.2 ± 4.28	0.933 ± 0.014

LR model	ROI uptake	72.4 ± 2.73	81.1 ± 5.99	60.7 ± 4.83	0.748 ± 0.037
	MCI pattern	74.8 ± 4.36	82.3 ± 2.49	66.8 ± 5.91	0.829 ± 0.036
	Connectome	82.3 ± 3.29	80.9 ± 3.14	84.3 ± 6.64	0.867 ± 0.043

Random forest	ROI uptake	70.8 ± 4.73	81.1 ± 3.75	59.3 ± 6.23	0.725 ± 0.045
	MCI pattern	73.1 ± 4.02	85.4 ± 2.86	61.4 ± 8.84	0.787 ± 0.032
	Connectome	76.2 ± 3.19	87.6 ± 2.99	62.9 ± 7.48	0.807 ± 0.031

The predictive performance of MCI participants was not involved in the training dataset.

**Table 3:** Predictive performance of different methods among MCI groups.

Classifier	Method	FPR	FNR
SVM	ROI uptake	33.3 ± 2.51	17.8 ± 1.72
	MCI pattern	32.9 ± 6.29	16.3 ± 4.46
	Connectome	18.8 ± 4.28	11.9 ± 3.17

LR model	ROI uptake	39.3 ± 4.83	18.9 ± 5.99
	MCI pattern	33.2 ± 5.91	17.7 ± 2.49
	Connectome	15.7 ± 6.64	19.1 ± 3.14

Random forest	ROI uptake	40.7 ± 6.23	18.9 ± 3.75
	MCI pattern	38.6 ± 8.84	14.6 ± 2.86
	Connectome	37.1 ± 7.48	12.4 ± 2.99

**Table 4:** The information of brain regions associated with MCI conversion.

Brain labels	Region	MNI coordinate (mm)		
		*X*	*Y*	*Z*
1	Precentral gyrus (left)	-38.65	-5.68	50.94
11	Inferior frontal gyrus (left)	-48.43	12.73	19.02
48	Lingual gyrus (right)	16.29	-66.93	-3.87
67	Precuneus (left)	9.98	-56.05	43.77
89	Inferior temporal gyrus (left)	-49.77	28.05	-23.17

**Table 5:** The predictive performance of the different methods in MCI conversion study.

Reference	Method	Conversion time (month)	sMCI (*n*)	pMCI (*n*)	Accuracy (%)	Sensitivity (%)	Specificity (%)	AUC
Young et al.	Gaussian process	36	96	47	65.0	66.0	64.6	0.767
Liu et al.	Independent component analysis and Cox model	36	108	126	68.8	57.1	82.4	0.736
Lange et al.	SPM *t*-test	36	77	31	/	/	/	0.832
Kengo et al.	Logistic regression	24	47	41	83	70	90	/
Lu et al.	Deep neural network	36	409	217	81.5	78.2	82.5	/
Pagani et al.	Independent components analysis	60	27	95	83.5	83.2	85.2	0.894
Proposed	Metabolic CPM	36	242	178	85.2	88.1	86.4	0.933

## Data Availability

The PET imaging data used to support the findings of this study may be released upon application to the ADNI database (http://adni.loni.usc.edu/).
